# Maternal gender discrimination and child emotional and behavioural problems: A population-based, longitudinal cohort study in the Czech Republic

**DOI:** 10.1016/j.eclinm.2022.101627

**Published:** 2022-08-27

**Authors:** Irena Stepanikova, Sanjeev Acharya, Alejandra Colón-López, Safa Abdalla, Jana Klanova, Gary L. Darmstadt

**Affiliations:** a Department of Sociology, University of Alabama at Birmingham, Birmingham, Alabama, USA; b RECETOX, Faculty of Science, Masaryk University, Brno, Czech Republic; c Department of Criminology, Sociology, and Geography, Arkansas State University, Jonesboro, Arkansas, USA; d Department of Pediatrics, Stanford University School of Medicine, Stanford, California, USA

**Keywords:** Gender discrimination, Mental health, Behavioural problems, Adverse childhood experiences, Child health, Adolescent health

## Abstract

**Background:**

Gender discrimination may be a novel mechanism through which gender inequality negatively affects the health of women and girls. We investigated whether children's mental health varied with maternal exposure to perceived gender discrimination.

**Methods:**

Complete longitudinal data was available on 2,567 mother-child dyads who were enrolled between March 1, 1991 and June 30, 1992 in the European Longitudinal Cohort Study of Pregnancy and Childhood-Czech cohort and were surveyed at multiple time points between pregnancy and child age up to 15 years. The Strengths and Difficulties Questionnaire (SDQ) was administered at child age 7, 11, and 15 years to assess child emotional/behavioural difficulties. Perceived gender discrimination was self-reported in mid-pregnancy and child age 7 and 11 years. Multilevel mixed-effects linear regression of SDQ scores were estimated. Mediation was tested using structural equation models.

**Findings:**

Perceived gender discrimination, reported by 11.2% of mothers in mid-pregnancy, was related to increased emotional/behavioural difficulties among children in bivariate analysis (slope = 0.24 [95% confidence interval (CI): 0.15, 0.32], *p<*0.0001) and in the fully adjusted model (slope = 0.18 [95% CI: 0.09, 0.27], *p<*0.0001). Increased difficulties were evident among children of mothers with more depressive symptoms (slope = 0.04 [95% CI: 0.03, 0.05], *p<*0.0001), boys (slope = 0.26 [95% CI: 0.19, 0.34], *p<*0.0001), first children (slope = 0.16 [95% CI: 0.09, 0.23], *p<*0.0001), and families under financial hardship (slope = 0.09 [95% CI: 0.04, 0.14], *p<*0.0001). Effects were attenuated for married mothers (slope-0.12 [95% CI: -0.22, -0.01], *p<*0.05]. Maternal depressive symptoms and financial hardship mediated about 37% and 13%, respectively, of the total effect of perceived gender discrimination on SDQ scores.

**Interpretation:**

Perceived gender discrimination among child-bearing women in family contexts was associated with more mental health problems among their children and adolescents, extending prior research showing associations with maternal mental health problems. Maternal depressive symptoms and, to a lesser extent, financial hardship both partially mediated the positive relationship between perceived gender discrimination and child emotional/behavioural problems. This should be taken into consideration when measuring the societal burden of gender inequality and gender-based discrimination. Moreover, gender-based discrimination affects more than one gender and more than one generation, extending to boys in the household even moreso than girls, highlighting that gender discrimination is everyone's issue. Further research is required on the intergenerational mechanisms whereby gender discrimination may lead to maternal and child mental health consequences.

**Funding:**

Bill and Melinda Gates Foundation; Ministry of Education, Youth and Sports, Czech Republic and European Structural and Investment Funds.

**Research in context**

**Evidence before this study**

Gender discrimination has been proposed as a stressor through which gender inequality negatively affects the health of women and girls. We searched Pubmed and Google Scholar databases for peer-reviewed publications in English and Czech before January 1, 2021, and screened the English abstracts of papers in Czech, using the key words “gender bias,” “sexism,” “discrimination,” “child mental health,” and “child emotional/behavioural problems” to identify empirical evidence on the relationship between gender discrimination and child mental health. We found emerging evidence suggesting that gender discrimination and sexism, like discrimination based on race and ethnicity, may lead to stress responses and adversely affect mental health; we showed previously using data from the European Longitudinal Cohort Study of Pregnancy and Childhood-Czech (ELSPAC-CZ) cohort that maternal perceived gender discrimination was associated with increased risk for maternal depression.

**Added value of this study**

We advanced our prior analysis to examine longitudinal effects of perceived maternal gender discrimination on the mental health of their children, and found that children born to the 11.2% of women who perceived mid-pregnancy that they had experienced gender discrimination had significantly (*p<*0.0001) higher levels of emotional/behavioural problems compared to the children born to women who did not perceive the experience of gender discrimination. Higher SDQ scores were associated with maternal depressive symptoms (*p<*0.0001), financial hardship in the family (*p<*0.0001), first children (*p<*0.0001), children of unmarried mothers (p = 0.022) and boys (*p<*0.0001), but not low birth weight. Maternal depressive symptoms and financial hardship significantly mediated the relationship between perceived gender discrimination and SDQ scores.

**Implications of all the available evidence**

This study using population-based, longitudinal data is the first, to our knowledge, to demonstrate a link between maternal perceived gender discrimination and adverse mental health outcomes for their children, and furthermore, that maternal depression linked to gender discrimination explained more than one-third of the adverse emotional/behavioural effects displayed by their children. Importantly, the effects of maternal gender discrimination were transmitted to boys and girls in households, with accentuated effects in boys, demonstrating the broader societal implications of gender discrimination for people of all genders that can occur over a relatively short period of time. While gender, racial, and other forms of discrimination appear to have some commonalities in effects, further research is needed to understand the pathways whereby gender discrimination becomes embodied and is translated into mental and physical effects; this knowledge will inform individual, household, societal, and structural interventions to prevent gender discrimination and its widespread effects on human health and well-being.

Alt-text: Unlabelled box

## Introduction

Emotional/behavioural problems have surged among children and youth in recent decades, with the largest increases for emotional problems and antisocial behaviour observed in high-income countries.^1^ The estimated prevalence of mental disorders among children and adolescents is 13.4% worldwide.^2^ Anxiety disorders are most common (6.5%), followed by disruptive disorders (5.7%), attention-deficit hyperactivity disorders (3.4%), and depressive disorders (2.6%).^2^ In Europe, 22% of children ages 6-12 years report symptoms that meet criteria for a mental disorder diagnosis.^3^

This research focuses on the role of gender inequality, manifest as perceived gender discrimination, in early origins of mental health disorders. Women face considerably higher risk of gender discrimination compared to men.^4^ In an overwhelming majority of societies, women occupy a subordinate position relative to men, holding less power and privilege. Beliefs, norms, and stereotypes widely held by men and women alike bolster social arrangements that disadvantage women. Ninety-one percent of men and 86% of women worldwide express pro-male biases in the areas of politics, education, economics, and physical integrity, e.g., intimate partner violence.^5^

*The Lancet* Series on Gender Equality, Norms and Health advanced gender discrimination as a mechanism through which gender inequality negatively affects the health of women and girls.^6^^,^^7^ Emerging evidence from cross-sectional research supports the argument that gender discrimination and sexism lead to poorer mental health outcomes.^8^ Population-based data from the European Longitudinal Cohort Study of Pregnancy and Childhood-Czech (ELSPAC-CZ) cohort indicated that perceived gender discrimination of child-bearing women was associated with significantly increased risk for maternal depressive symptoms.^9^

Implications of maternal exposure to gender discrimination for the child are largely unknown but studies report adverse mental health outcomes for children whose parents were exposed to racial or ethnic discrimination and discrimination due to any cause.^10^ Discrimination presents a special concern if it takes place during pregnancy and early life when the child's nervous system is immature and undergoing rapid development. Mother's exposure to discrimination as a stressor may lead to physiological stress responses, with stress hormones crossing the placenta and potentially dysregulating fetal neuro-endocrine development. Additionally, psychosocial stressors caused by exposure to discrimination may undermine parents’ mental health,^8^^,^^11^ compromise parent-child bonding, and lead to more hostile parenting,^12^ all factors in child emotional and behavioural problems.

**Figure 1:**
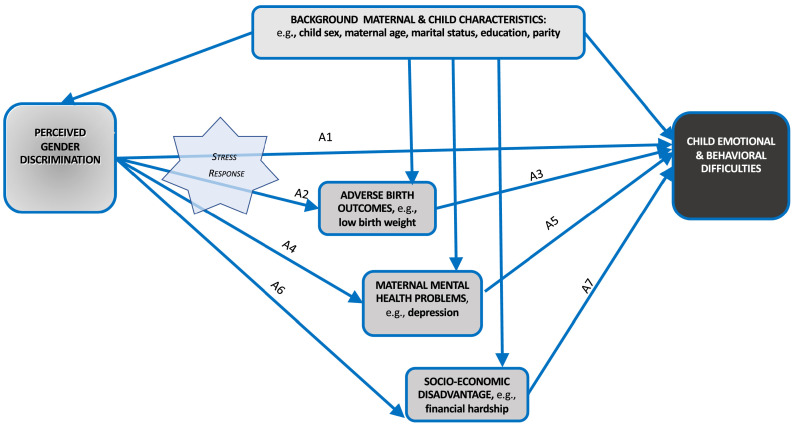
**Conceptual model of key factors in the association of maternal perceived gender discrimination and child emotional/behavioural difficulties**.

Our conceptual model for this study centers on gender discrimination as a stressor and acknowledges the importance of mutual influences within parent-child dyads.^13^, ^14^, ^15^ Exposure to perceived discrimination causes stress responses in mothers and children, leading to potential dysregulation in child psychological functioning directly though biological pathways and indirectly through mediated mechanisms. The direct effect (Figure 1, Arrow A1) mainly represents effects of stress hormone exposure on fetal neurodevelopment, including changes in structure-function of the brain and neuro-endocrine system, and epigenetic processes. Such changes are especially likely during the fetal stage, when foundations of brain architecture are being laid, but cannot be ruled out as occurring during infancy and later childhood. The brain remains remarkably plastic throughout the entire period of childhood and adolescence and sensitive periods for development open up at different stages along this time course.

Arrows A2-A5 in our conceptual model represent indirect effects of gender discrimination through adverse birth outcomes (A2, A3) and maternal psychological problems (A4, A5) as mediators (Figure 1). These mediators were selected as plausible mechanisms linking gender discrimination to child mental health. Prior evidence indicates that they correlate with child psychopathology^16^, ^17^, ^18^ and gender discrimination.^19^, ^20^, ^21^, ^22^ Individuals who report racial discrimination, for instance, face an increased risk for major depression^19^ and maternal depression is an established risk factor for emotional problems in children.^14^^,^^17^ Racial discrimination is linked to adverse birth outcomes,^20^ known predictors of child psychopathology.^23^ Another proposed mediator is socio-economic disadvantage (A6, A7), represented by financial hardship.^24^ Gender discrimination commonly happens in workplaces, adversely affecting women's access to job opportunities, training, and career advancement. For instance, women may perceive discrimination when they are rejected as job candidates in favour of a male, passed up for a promotion, or when they know of pay differences between male and female in the company who perform the same job. When gender discrimination happens in the job market or workplace, it has a potential to negatively affect the socio-economic prospects of the family and have adverse implications for the children.^16^^,^^25^

Here we build on our prior analysis showing increased risk for depressive symptoms in child-bearing women associated with perceived gender discrimination,^9^ and examine longitudinal effects on their children. Using the proposed conceptual model, we derive the following hypotheses to be tested with prospective data: 1) Maternal perceived gender discrimination is linked to child emotional/behavioural problems, and 2) The relationship between maternal perceived gender discrimination and child emotional/behavioural problems is mediated by low birth weight, maternal depression, and financial hardship.

## Methods

### Study design

This longitudinal, observational cohort study utilised data from the population-based, longitudinal ELSPAC-CZ cohort in the Czech Republic.^26^ ELSPAC studies were initiated by the World Health Organization to investigate maternal and child health in several European countries, including the influence of biological, psychological, social, economic and environmental factors on the health of children and adolescents. Families were followed longitudinally for two decades, during pregnancy and through age 18 years of the children.

### Sample

**Figure 2:**
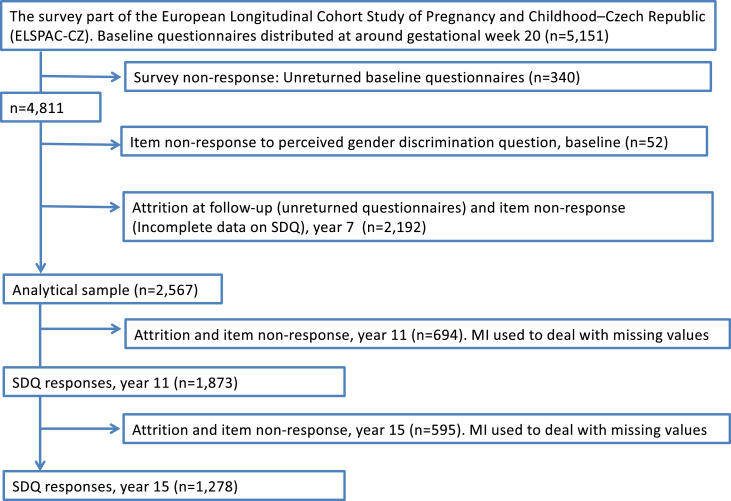
**Flow chart showing inclusion and exclusion of respondents due to non-response**. MI, multiple imputation; SDQ, Strengths and Difficulties Questionnaire

ELSPAC-CZ was conducted in Brno, a large metropolis, and Znojmo, a small nearby town to represent urban and rural populations, respectively. The study population was defined as all pregnancies and births in these two regions of the Czech Republic in 1991 and 1992. Over 99% of the residents in both regions consisted of the Czech ethnic group in 1991-92.^26^ Participants were recruited in mid-pregnancy. Eligibility criteria included residence in one of these two cities and an expected date of delivery between March 1, 1991 and June 30, 1992. Mothers were enrolled between the ultrasound examination at week 20 of pregnancy and the birth. Eligible mothers received information about the study from their obstetricians who forwarded contact information of women who were interested in the study to the study team. These women were officially invited by mail to participate in the study. Detailed information on the study methodology and cohort profile are available elsewhere.^26^ Health records were collected for 7589 births: 96% of all eligible births. A subsample (*n =* 4,811) were surveyed at 20 weeks gestation (henceforth called mid-pregnancy, identified as described above) and among these, 4630 women (96.2%) completed a baseline questionnaire which included a measure of perceived discrimination. Follow-up questionnaires were mailed to participants at 15 time points between the child's birth and age 19 years; intervals between successive mailings ranged from six months to four years. Child mental health symptoms, our main outcome, was assessed using the Strengths and Difficulties Questionnaire (SDQ) administered to participating mothers at child ages 7, 11, 15, and 18 years. We did not use child-reported data because they were not consistently available across all ages. SDQ data were available for 2619 7-year-old children, which represents 54.4% of the 4811 in the original sample. Among the mothers of these children, 98.0% (*n =*  2,567) had complete baseline data on perceived gender discrimination collected in mid-pregnancy and thus were included in our final analytical sample. Among these, 1,873 also completed the SDQ questionnaire at child age 11 and 1,278 at child age 15 (Table S1); to account for the missing data, we used information avaialable in the final analytical sample and applied multiple imputation methodology. Figure 2 summarises inclusion and exclusion of respondents due to non-response.

### Measures

Delivery characteristics were extracted from medical documentation at the time of birth; the remaining measures were obtained by maternal self-report on written surveys administered at the times indicated below.

Scores from the SDQ, used as the primary outcome, are a validated measure of child and adolescent symptoms indicating emotional and behavioural problems, including hyperactivity, emotional symptoms, conduct problems, and peer problems. SDQ was mother-reported at ages 7, 11, and 15 years. Since slightly different response categories were used in some waves, we calculated z-scores within each wave. The resulting standardised scores were used to achieve comparability across years. We did not use SDQ data at child age 18 years due to a relatively large number of missing cases.

Maternal depressive symptoms, low birth weight, and financial hardship were used for the purposes of mediation analysis. Maternal depressive symptoms were measured using the Edinburgh Postnatal Depression Scale (EPDS), which has been validated for assessment of depression during pregnancy and the first year postpartum.^27^ Controlling for maternal depression was important because deprerssed mothers may report more discrimination and rate their children's behaviour as more problematic.^28^^,^^29^ The EPDS consists of ten items rated on a four-point scale (0 = “Never”, 3 = “Most of the time”), such as “I have felt sad or miserable.” The scale was administered at baseline (mid-pregnancy), childbirth, and child ages 6 and 18 months, and 3, 5, 7, and 11 years. Low birthweight (<2,500 grams) was extracted from medical documentation to represent adverse birth outcomes.

For the financial hardship scale, women reported how difficult it was to provide for their family food, clothing, heating, rent/mortgage payments, and provisions for their child at ages 3, 5, and 7 years. Each item was rated on a four-point scale (0 = “Not difficult”, 3 = “Very difficult”). The mean across items was used to represent overall financial hardship.

Perceived gender discrimination, the main predictor, was assessed in mid-pregnancy and when the children were ages 7 and 11 years. Respondents were asked, “Would you say that during the past twelve months, someone treated you unfairly because of your gender?” The measure captures respondents’ perceptions of whether they have experienced any gender discrimination (“yes”) vs. no gender discrimination (“no”). Self-reported perceived discrimination is widely used in observational studies of discrimination based on race, ethnicity, language, religion, and sexual identity.^30^^,^^31^

Confounders included social support at baseline, which was measured with a scale consisting of five items: 1) “How many people can you talk to about your personal problems?”, 2) “How many people talk to you about their inner feelings?”, 3) “When you need to make an important decision, with how many people can you discuss it?”, 4) “How many people in your family would lend you 1000 Czech crowns if you needed them?”, and 5) “How many people in your family would help you in the time of need?” The mean was used to indicate the average number of supportive individuals in respondents’ lives. Additional confounders included demographic and delivery characteristics. Maternal marital status (single, married, divorced/separated, widow) and maternal education (in years at baseline) were assessed; age was recorded at the birth of the focal child. Delivery characteristics extracted from medical documentation included child sex (male vs. female), singleton vs. multiple birth, and number of childbirth complications. To proxy parity, pregnant women reported whether they already had children. Pregnancy with the first child was coded as 1; second and higher-order children were coded as 0. Confounders were selected because they correlate with psychological issues among children.

### Statistical analysis

Descriptive statistics and bivariate tests of the relationships between SDQ and other analytical variables were obtained. Next, the proposed hypotheses were tested using multi-level mixed-effects linear regression, also known as linear mixed error-component models. They are a type of hierarchical linear modeling that accounts for time and correlations among the repeated observations nested within respondents. Here, the model accounts for both within-person (i.e., within mother) and across-mother variability in the final estimates. Standardised SDQ scores served as the primary outcome; others were secondary outcomes. Perceived gender discrimination was the main explanatory variable. Time was coded as years since baseline. For time-varying predictors that were not collected in all survey waves, the nearest value for that variable in time was assumed. For example, perceived gender discrimination at 5 years after delivery (not collected) was assumed to be the same as perceived gender discrimination at 7 years after delivery (collected). This technique allowed us to use all available predictor data and also allowed for covariates which were assessed in selected waves only to vary over time. This was done with all predictors so that all waves in which SDQ data were collected could be included in the analyses. Sensitivity analyses included testing for interactions between perceived discrimination and each covariate. We also assessed whether there were non-linear effects of time and maternal depression, and whether the slope of over-time change in SDQ scores varied between exposed and non-exposed children. Mediation analysis was conducted using structural equation models (SEM) as described by Gunzler et al.^32^ The purpose was to test whether maternal perceived gender discrimination and child emotional/behavioural problems were mediated by low birth weight, maternal depression, and financial hardship. Direct, indirect, and total effects were estimated using bootstrapping with 1,000 replications. Significance testing of indirect effects of perceived gender discrimination on SDQ through potential mediators was performed using medsem Stata package. The medsem is a post-estimation command to test mediation hypotheses using Baron and Kenny's approach modified by Iacobucci et al. as described by Mehmetoglu.^33^, ^34^, ^35^ The medsem command also utilises an alternative approach proposed by Zhao et al.^36^ to test the indirect effects after computing mediation models with the sem command in Stata, as described by Mehmetoglu.^33^ We used both Sobel and Monte Carlo methods to test the significance of indirect effects. To account for the loss to follow-up and missing data, we used multiple imputations with chained equations, i.e., sequential regression multivariate imputation.^37^ Imputations were conducted on the wide data version (one observation per individual). We imputed SDQ at 11 and 15 years, but not at 7 years, to have a consistent sample size. Unless otherwise stated, the results presented here are based on the data after multiple imputations. Analyses were conducted using Stata statistical software, version 16.1 (StataCorp LLC, College Station, Texas, USA).

### Ethical approval

ELSPAC-CZ was approved by the Scientific Committee of Masaryk University. All participants provided written informed consent. The study was also approved by the Stanford University Institutional Review Board protocol # 42971.

### Role of the funding source

The funders of the study had no role in study design, data collection, data analysis, data interpretation, or writing of the report. All authors had full access to the data in the study and the corresponding author had final responsibility to submit the paper for publication.

## Results

### Sample characteristics

At baseline, participating women were mostly married (89.4%, *n =* 2,295 of 2,567) and had a mean age at childbirth of 25.9 years with an average of 12.3 years of education (Table 1). Women reporting perceived gender discrimination were significantly older (26.5 vs 25.8 years, *p =* 0.019) and more educated (12.8 vs 12.2 years, *p<*0.001), and had higher levels of financial hardship (0.61 vs 0.49, *p =* 0.013) and lower levels of social support (3.09 vs 3.26, *p =* 0.005) than women who did not report perceived gender discrimination. Alpha coefficients for reliability based on the pooled sample were 0.68 for the financial hardship scale, 0.78 for the social support scale, and 0.87 for the maternal depression scale, indicating acceptable levels of reliability. For over a third of women (38.9%, *n =* 999 of 2567), the index child was their first child. Most births were singleton infants (98.2%, *n =* 2521 of 2567) (compared to multiple births), with normal birthweight (95.8%, *n =* 2459 of 2567). Male children (51.8%, *n =* 1330 of 2567) were in a slight majority. There were no differences in pregnancy and childbirth characteristics between women with and without perceived gender discrimination, except that women with perceived gender discrimination had a lower rate of low birthweight (1.6% vs 4.5%, *p =* 0.020).

**Table 1:** Characteristics of the sample of Czech Republic mothers and their children at baseline, shown by mothers who did and did not report perceived gender discrimination at mid-pregnancy.^a^

	Gender discrimination							All		
	Yes (*n = *288)			No (*n = *2279)			*p*-value^b^	(*n = *2567)		
Variable (Range)	Mean or % SE	95% CI		Mean or % SE	95% CI			Mean or % SE	95% CI	
SDQ^c^ score at 7 years (0-1.5)	0.51	0.48	0.54	0.45	0.44	0.46	<0.001	0.45	0.44	0.46
	0.01			0.005				0.004		
Perceived gender discrimination (%)								11.22	10.76	12.44
Depressive symptoms (0-29)	8.29	7.71	8.87	6.11	5.93	6.29	<0.001	6.35	6.18	6.53
	0.29			0.09				0.09		
*Socio-demographic characteristics (mean)*										
Age at birth, years	26.53	25.97	27.09	25.82	25.62	26.02	0.019	25.90	25.72	26.09
	0.28			0.10				0.09		
Education, years	12.75	12.44	13.06	12.20	12.10	12.30	<0.001	12.26	12.17	12.35
	0.16			0.05				0.04		
Financial hardship (scale of 0-3)	0.61	0.51	0.70	0.49	0.46	0.52	0.013	0.50	0.48	0.53
	0.04			0.01				0.01		
Social support (scale of 0-5)	3.09	2.98	3.20	3.26	3.22	3.30	0.005	3.24	3.20	3.28
	0.06			0.02				0.02		
*Marital status* (% married)	87.66	83.82	91.50	89.65	88.39	90.91	0.303	89.43	88.23	90.63
*Characteristics of pregnancy and childbirth*										
First child (%)	36.46	30.86	42.05	39.24	37.23	41.24	0.361	38.93	37.04	40.81
Low birth weight (%)	1.55	0.06	3.16	4.54	3.66	5.42	0.020	4.20	3.41	5.00
Boy child (%)	53.82	48.02	59.61	51.60	49.54	53.65	0.478	51.85	49.91	53.78
Singleton delivery (%)	99.34	98.31	100.37	98.01	97.43	98.60	0.122	98.16	97.64	98.69
Birth complications (0-12 complications)	0.37	0.29	0.46	0.31	0.28	0.34	0.176	0.32	0.29	0.35
	0.04			0.01				0.01		

a Means and percentages were obtained using the dataset treated with multiple imputations with chained equations; thus, SE (standard error) and 95% CI (confidence interval) are presented as measures of uncertainty.

b p-value is obtained from bivariate mixed models of each variable with baseline gender discrimination.

c SDQ = Strengths and Difficulties Questionnaire.

Perceived gender discrimination rates ranged from 10.6% to 11.2% across survey years (11.2%, *n =* 288 of 2295 in mid-pregnancy; 10.6%, *n =* 272 of 2295 at age 7 years; 10.6%, *n =* 273 of 2295 at age 11 years) (Table 2).

**Table 2:** Maternal perceived gender discrimination and child strength and difficulties questionnaire (SDQ) scores.

	Perceived gender discrimination							All		
	Yes (*n=*288)			No (*n=*2279)			*p*-value	(*n=*2567)		
Variable (Range)	Mean SE^a^	95% CI^b^		Mean SE	95% CI			Mean or % SE	95% CI	
Perceived gender discrimination (%)										
Mid-pregnancy								11.22	10.76	12.44
								0.006		
7 years	24.72			8.80				10.59	9.37	11.80
	0.025			0.006				0.006		
11 years	23.39			9.00				10.62	9.13	12.10
	0.024			0.007				0.007		
SDQ score (mean)										
7 years (range 0-1.5)	0.51	0.48	0.54	0.45	0.44	0.46	<0.001	0.45	0.44	0.46
	0.01			0.005				0.004		
11 years (range 0-2.21)	0.74	0.70	0.79	0.67	0.66	0.69		0.68	0.67	0.69
	0.02			0.007				0.006		
15 years (range 0-1.4)	0.40	0.34	0.45	0.37	0.36	0.38		0.37	0.36	0.38
	0.02			0.005				0.005		

a SE = Standard error.

b CI = Confidence interval.

### SDQ score trajectory by gender discrimination

**Figure 3:**
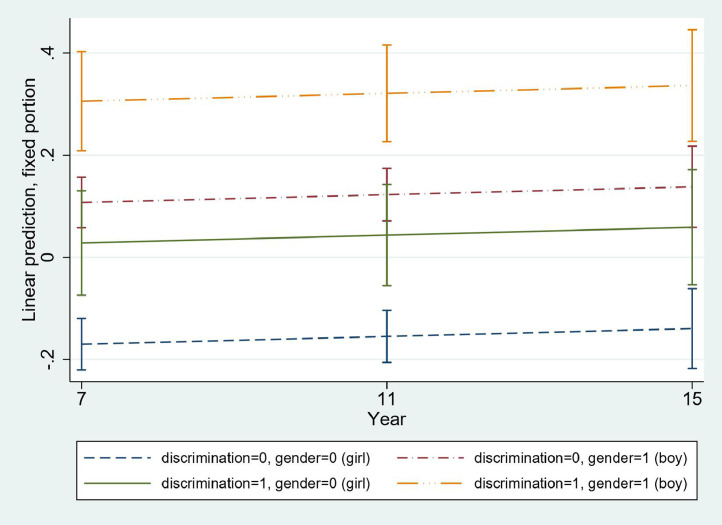
**Trends over time in adjusted predictions (with 95% confidence intervals) of Strengths and Difficulties Questionnaire scores of boy and girl children of mothers who did and did not perceive gender discrimination**.

The alpha coefficient for reliability (0.75) for SDQ scores based on pooled data indicated acceptable reliability. Mean SDQ scores were similar before and after multiple imputations (Table 2, Table S1), and showed an increase between ages 7 and 15 years (Figure 3), with higher scores for children exposed to perceived gender discrimination during pregnancy. The mean difference in SDQ scores between exposed and non-exposed children before adjustment for covariates ranged from 0.03 to 0.07 (Table 2).

Consistent with our hypothesis, the relationship between perceived gender discrimination and SDQ scores persisted after adjustment for time, auto-correlations, and maternal- and child-level covariates in multilevel mixed-effects linear regression models (slope = 0.18, 95% CI 0.09-0.27; *p<*0.0001, Table 3, model 2). The over-time trend in SDQ scores between ages 7 and 15 years increased linearly, with similar slopes for boys and girls and statistically different predicted SDQ scores for boy and girl children of mothers who did or did not have experience of perceived gender discrimination (Figure 3). Among covariates in the regression models for the relationship between perceived gender discrimination and SDQ scores, maternal depressive symptoms (slope = 0.04, 95% CI 0.03-0.05; *p<*.0001) and financial hardship in the family (slope = 0.09, 95% CI 0.04-0.14; *p<*0.0001) were linked to higher SDQ scores (Table 3, model 2). First children (slope = 0.16, 95% CI 0.09-0.23; *p<*.0001) and boys (slope = 0.26, 95% CI 0.19-0.34; *p<*.0001) showed higher SDQ scores, while children of married mothers (slope = -0.12, 95% CI -0.22 to -0.01; *p =* 0.022) had lower SDQ scores. Time, maternal age, education, social support, age at birth, and childbirth complications were not associated with SDQ scores.

**Table 3:** Multi-level mixed-effects linear regression model outputs for mother-reported child Strengths and Difficulties Questionnaire scores in relation to maternal perceived gender discrimination (*N=*2,567).

Variables	Model 1				Model 2			
	b	*p*-value	95% CI^a^		b	*p*-value	95% CI	
Perceived gender discrimination	0.24	<0.0001	0.15	0.32	0.18	<0.0001	0.09	0.27
Time	−0.00004	0.855	−0.0005	0.0004	0.0003	0.483	−0.0005	0.001
Depressive symptoms					0.04	<0.0001	0.03	0.05
*Socio-Demographic Characteristics*								
Financial hardship					0.09	<0.0001	0.04	0.14
Education					0.009	0.227	−0.005	0.02
First child					0.16	<0.0001	0.09	0.23
Low birth weight					0.01	0.890	−0.17	0.19
Boy child					0.26	<0.0001	0.19	0.34
Singleton delivery					0.06	0.691	−0.27	0.41
Marital status^b^								
Married					−0.12	0.022	−0.22	−0.01
Social support					−0.02	0.272	−0.05	0.01
Age at birth					0.004	0.276	−0.003	0.01
Birth complications					0.04	0.185	−0.02	0.09

a CI = Confidence interval.

b Reference category = Single/widowed/divorced/separated.

Sensitivity analyses (available upon request) revealed no statistically significant non-linearity for time and no interactions between gender discrimination and any of the covariates. A significant quadratic effect of maternal depression was present, indicating that as mothers are more depressed, the effect of depression on child SDQ is diminished (unsquared term, slope = 0.05, 95% CI 0.04-0.06; *p<*0.0001; squared term, slope = -0.001, 95% CI -0.002–0.0002; *p<*0.05).

### Mediation between perceived gender discrimination and SDQ scores

The structural equation models showing the direct, indirect and total effects of perceived gender discrimination are presented in Table 4. The relationship between perceived gender discrimination and SDQ was mediated by maternal depression, which was significantly linked to SDQ (statistics above) and gender discrimination. Maternal depression accounted for 37% of the total effect of perceived gender discrimination [ratio of the indirect effect to the total effect (RIT) = 0.369, Table 5], and explained 2.4% of the total variance (direct effect slope 0.21, 95% CI 0.10-0.31, *p<*0.0001; indirect effect slope 0.12, 95% CI 0.09-0.15, *p<*0.0001; total effect slope 0.33, 95% CI 0.22-0.44, *p<*0.0001) (Table 4). Financial hardship correlated with SDQ (results above) and perceived gender discrimination and mediated 13% of the total effect of perceived gender discrimination, accounting for 1.5% of the total variance (direct effect slope 0.21, 95% CI 0.10-0.31, *p<*0.0001; indirect effect slope 0.03, 95% CI 0.01-0.04, *p<*0.0001; total effect slope 0.24, 95% CI 0.13-0.34, *p<*0.0001). The interpretation is that maternal depression and financial hardship significantly mediate the association between gender discrimination and child emotional/behavioral problems. Low birth weight was not linked to perceived gender discrimination (*p =* 0.890).

**Table 4:** Summary of total, direct, and indirect effects of maternal perceived gender discrimination on child Strengths and Difficulties Questionnaire scores based on Structural Equation Modelling mediation analysis (*N = *2,567).^a^

Variable	Direct Effect				Indirect Effect^b^				Total Effect			
	b	*p*-value	95% CI^c^		b	*p*-value	95% CI		b	*p*-value	95% CI	
	Mediator: Depressive symptoms											
Perceived gender discrimination	0.21	<0.0001	0.10	0.31	0.12	<0.0001	0.09	0.15	0.33	<0.0001	0.22	0.44
	Mediator: Financial hardship											
Perceived gender discrimination	0.21	<0.0001	0.10	0.31	0.03	<0.0001	0.01	0.04	0.24	<0.0001	0.13	0.34

a All models are adjusted for socio-demographic characteristics (financial hardship, education, first child, low birth weight, boy child, singleton delivery, marital status, social support, age at birth, birth complications, time) (see Table 1).

b Indirect effect in this model refers to the indirect path of perceived gender discrimination through depressive symptoms and financial hardship.

c CI  =  Confidence interval.

**Table 5:** Significance testing of indirect effects of maternal perceived gender discrimination on child Strengths and Difficulties Questionnaire scores mediated through maternal depressive symptoms and financial hardship (*N = *2567)

Estimates	Depressive symptoms		Financial hardship	
	Sobel	Monte Carlo	Sobel	Monte Carlo
Indirect effect	0.038	0.038	0.009	0.009
Standard error^a^	0.004	0.004	0.002	0.002
z-value	8.559	8.558	4.147	4.133
p-value	<0.0001	<0.0001	<0.0001	<0.0001
Confidence interval	[0.029; 0.046]	[0.029; 0.047]	[0.005; 0.014]	[0.005; 0.014]
RIT^b^	0.369		0.127	

a Standard errors were obtained after bootstrapping the same number of repetitions as the observations. Results were obtained using the medsem command in Stata developed by Mehmetoglu.^33^

b RIT = Ratio of the indirect effect to the total effect.

## Discussion

This prospective observational study followed mother-child pairs from mid-pregnancy until age 15 years and evaluated the relationship between maternal perceived gender discrimination and child emotional/behavioural problems. We found that the children of women who perceived that they had experienced gender discrimination between pregnancy and child age 11 years had more emotional/behavioural problems between ages 7 and 15 years compared to the children of women who had not experienced perceived gender discrimination. First children and boys displayed greater emotional/behavioural problems, while children of married mothers had fewer such problems. Importantly, maternal depressive symptoms and financial hardship in the family were linked to higher child SDQ scores, and furthermore were found to significantly mediate the relationship between maternal gender discrimination and child emotional/behavioural problems.

To our knowledge, this represents the first study – using robust population-based, longitudinal data – to document a link between maternal gender discrimination and adverse mental health outcomes for their children. Specifying the link between mother's experience of gender discrimination and child psychopathology extends prior research, which documents that mental health problems can develop among victims of gender discrimination.^9^^,^^38^ Consistent with the concepts of inter-generational transmission of health risk,^39^ as seen, for example, following adverse childhood experiences,^14^^,^^15^^,^^40^ our findings indicate that gender inequality in a society experienced individually as gender discrimination may have negative health implications for the victim's offspring. This should be taken into consideration when measuring the societal burden of gender inequality and gender-based discrimination – an important area for future investigation.

This study evaluated several potential mechanisms through which maternal gender discrimination may link to child mental health. We found that the statistical effect of gender discrimination was strongly mediated through maternal depression and less so by family financial difficulties but not through low birth weight of the child. Mechanisms through which gender inequality translates into health disadvantages for children are poorly understood and require further research that encompasses measures of biological processes as well as social determinants and their interactions with biology.^6^^,^^41^ These processes have been described as “embodiment,”^6^^,^^41^ the process of translation of stressors into stress and thence into biological pathways toward disease. For gender discrimination more specifically, we know of no research on embodiment mechanisms linking maternal discrimination exposure to child health, though previous studies considering perceived racial and ethnic discrimination among mothers link it to adverse health outcomes among their children through a mechanism of maternal depression.^42^ While there appear to be similarities in effects of discrimination based on gender, race, and ethnicity, further research is needed to better understand ways in which these and other stressors become translated into adverse mental and physical health. Understanding similarities and differences in pathways for the effects of different forms of discrimination is an important step in informing effective individual, household, societal, and structural solutions to prevent, mitigate and treat the consequences of discrimination broadly.

This study represents a significant contribution to the literature on the impact of gender inequality and gender-based discrimination by clearly illustrating that it is not an issue that affects one gender or one generation but extends to all children – even moreso in boys than girls – in the household, presumably due in part to its effects on, or reflections of, the environment in which children grow up, leading to broader societal effects on people of all genders and across generations. The larger direct effect that we found is better considered a residual effect that is yet to be explained because of the possible presence of unmeasured common causes or mediators. Illustrating that gender discrimination is everyone's issue and not only a women's issue can help to alter the conversation at household and community levels where gender roles and norms are constantly negotiated,^6^ as well as at policy level and regarding structural-institutional factors which create the ecosystem in which gender discrimination occurs. Gender discrimination is everyone's issue and addressing gender discrimination and shaping gender norms to promote equality requires interventions involving a broad range of stakeholders and utilising a variety of levers at multiple levels of society.^43^, ^44^, ^45^

More specifically, interrupting the pathways that extend the mental health impact from mothers to their children can be one way of limiting the adverse impact of gender-based discrimination. One approach is to address the causes of mental health distress in clinical settings. The discourse around addressing the mental health impact of discrimination in clinical settings is limited thus far to the context of racial discrimination. Important lessons, however, can be drawn from this literature. Concepts like cultural safety where practitioners examine their own attitudes, cultural humility that encourages practitioners’ self-reflection, and cultural narrative where practitioners attend carefully to their patients’ stories are advocated to help practitioners provide more holistic care for mental health problems that supports and empowers patients to take control of their aggravating circumstances.^46^ Extending such concepts to gender-based discrimination may be similarly impactful, but can be challenging given the complexity that gender discrimination often takes place in the context of close interpersonal relationships with norms that include unfair distribution of household labour and decision-making.^6^^,^^47^ Interestingly, we have found in previous research that while social support was an important factor in development of depression among women who perceived gender discrimination,^9^ while here we found that social support was not associated with mental health problems in their children. Another challenge is that prevalent gender stereotyping in the community may prevent women with perceptions of discrimination to voice their concerns or acknowledge such perceptions as a potential cause of their mental health issues.

Addressing gender-based discrimination remains a key policy approach for preventing its adverse impacts.^45^ A barrier to women's economic participation and opportunity, discrimination can adversely affect the overall financial situation of the family through segregation of women into less desirable jobs, barriers to women's career advancement, part-time employment, precarious work, and unemployment. Importantly, mother-reported financial hardship emerged in our study as an important mechanism through which gender discrimination translated into adverse effects on child mental health. Women are at higher risk for economic difficulties and poverty compared to men in many societies throughout the world. In the Czech Republic, women are overrepresented in the public sector, where jobs tend to be less lucrative. In contrast, the Czech non-public sector offers higher earnings on average but women tend to earn alarmingly less compared to men.^48^ A substantial gender gap in earnings remains even after considering job characteristics, workers’ skills, and training, suggesting a considerable extent of gender discrimination. In a worldwide ranking of women's economic participation and opportunity in 2014, the Czech Republic placed in the bottom third,^49^ lower than many counterparts with similar economic levels. Major strides have been taken in terms of policy development to eliminate all forms of gender-based discrimination against women in the Czech Republic.^50^ Policy change to promote gender equality remains focused, however, on overt discrimination in institutional settings and may not exert influence in situations where perceptions of discrimination arise from more subtle social interactions within the community or family; unpaid care work and unfair household division of labour can be such sources. Therefore, a wider array of policy responses than currently considered may be required to address this multi-faceted problem.

A chief strength of this study is the population-based, longitudinal design which enabled us to establish the temporal association between perceived experience of gender-based discrimination and subsequent mental health outcomes for the children of the women who experienced the discrimination. Standard, validated scales were used to measure the mental health outcomes.

Limitations to note concern the fact that not all potential confounders were measured and some of the scales consisting of multiple items differed across years in wording and numbers of included items. To account for these differences and for missing values, and to make indicators comparable, we used statistical standardisation. We relied on answers to a single question about the presence of perceived gender discrimination. While this enabled powerful insights to be gained, we lacked biological correlates to corroborate that this perceived stressor led to biological stress responses, and we lacked additional data to explore pathways whereby the stressor became embodied in the biology of the women who experienced perceived gender discrimination and in their children. Another potential factor not examined here is cohabitation. At the time the ELSPAC survey was conducted, however, cohabitation not oriented toward marriage was rare among childbearing couples.^51^ We also lacked sufficient data on socio-demographic and clinical characteristics of fathers. Finally, the generalisability of the findings outside of the Czech Republic is unknown, but it is likely that they apply to other regions inside the Czech Republic. The demographic profile of the ELSPAC-CZ sample is similar to the entire Czech population. The proportion of the population of non-Czech ethnicities is low and the sample does not contain enough of these respondents. Thus, the findings are considered applicable to people of Czech ethnic group residing in the Czech Republic. Gender discrimination is pervasive globally but the social and political context of the study is unique, making it important to examine gender discrimination and child issues in other ethnic groups and other regions.

In conclusion, this study demonstrates that gender-based discrimination could have far reaching consequences for the health and well-being of societies – extending within families across generations and genders. It is not a women's issue only but can be transmitted inter-generationally and extend within households to people of all genders within a relatively short time span. These amplified effects are a cause for concern in a society where progress to implement gender equality policies can be slow. Efforts to address gender-based discrimination in all its manifestations are critical for preventing its adverse effects. While important lessons can be learned from literature on racial and ethnic discrimination, further research should specifically examine potential pathways and other direct and indirect outcomes of gender-based discrimination in the Czech Republic and beyond, given its ubiquitous and varied nature.

## Contributors

I.S. and G.L.D. conceptualised the paper; S.A., I.S. and J.K. managed data curation; formal analysis was conducted by S.A and I.S.; G.L.D. acquired funding; investigation was conducted by S.A and I.S.; methodology was devised by I.S., S.A., and S.Abdalla; G.L.D. managed project administration and resources; G.L.D. and I.S. provided supervision; S.Abdalla and A.C.L. validated study findings;: I.S. and S.A. led data visualisation; I.S., G.L.D., and S.Abdalla led writing of the original draft; all authors contributed to writing review and editing. All authors contributed intellectual content and approved the final draft for publication. All authors had full access to the data in the study and take responsibility for the integrity and accuracy of the data analysis; I.S. and S.A. verified the underlying data.

## Data sharing statement

Data may be requested at www.elspac.cz.

## Declaration of interests

The authors declare no conflicts of interest.

## Appendix Supplementary materials

Image, application 1

## References

[1] Collishaw S. (2015). Annual research review: secular trends in child and adolescent mental health. J Child Psychol Psychiatry Allied Discip.

[2] Polanczyk G V, Salum GA, Sugaya LS, Caye A, Rohde LA. (2015). Annual research review: a meta-analysis of the worldwide prevalence of mental disorders in children and adolescents. J Child Psychol Psychiatry Allied Discip.

[3] Husky MM, Boyd A, Bitfoi A (2018). Self-reported mental health in children ages 6–12 years across eight European countries. Eur Child Adolesc Psychiatry.

[4] Ayalon L. (2014). Perceived age, gender, and racial/ethnic discrimination in Europe: results from the European Social Survey. Educ Gerontol.

[5] Homan P. (2019). Structural sexism and health in the United States: a new perspective on health inequality and the gender system. Am Sociol Rev.

[6] Heise L, Greene ME, Opper N (2019). Gender inequality and restrictive gender norms: framing the challenges to health. Lancet.

[7] Weber AM, Cislaghi B, Meausoone V (2019). Gender norms and health: insights from global survey data. Lancet.

[8] Vines AI, Ward JB, Cordoba E, Black KZ. (2017). Perceived racial/ethnic discrimination and mental health: A review and future directions for social epidemiology. Curr Epidemiol Reports.

[9] Stepanikova I, Acharya S, Abdalla S, Baker E, Klanova J, Darmstadt GL. (2020). Gender discrimination and depressive symptoms among child-bearing women: ELSPAC-CZ cohort study. EClin Med.

[10] Tran AGTT. (2014). Family contexts: parental experiences of discrimination and child mental health. Am J Community Psychol.

[11] Khan M, Ilcisin M, Saxton K. (2017). Multifactorial discrimination as a fundamental cause of mental health inequities. Int J Equity Health.

[12] Bécares L, Nazroo J, Kelly Y. (2015). A longitudinal examination of maternal, family, and area-level experiences of racism on children's socioemotional development: patterns and possible explanations. Soc Sci Med.

[13] Pearlin LI, Menaghan EG, Lieberman MA, Mullan JT. (1981). The stress process. J Health Soc Behav.

[14] Kang NR, Kwack YS, Song J-K (2022). The intergenerational transmission of maternal adverse childhood experiences on offspring’s psychiatric disorder and the mediating role of maternal depression: results from a cross sectional study. Clin Child Psychol Psychiatry.

[15] Thomas-Argyriou JC, Letourneau N, Dewey D, Campbell TS, Giesbrecht GF. (2021). The role of HPA-axis function during pregnancy in the intergenerational transmission of maternal adverse childhood experiences to child behavior problems. Dev Psychopathol.

[16] Deighton J, Lereya ST, Casey P, Patalay P, Humphrey N, Wolpert M. (2019). Prevalence of mental health problems in schools: poverty and other risk factors among 28 000 adolescents in England. Br J Psychiatry.

[17] Priel A, Djalovski A, Zagoory-Sharon O, Feldman R. (2019). Maternal depression impacts child psychopathology across the first decade of life: oxytocin and synchrony as markers of resilience. J Child Psychol Psychiatry.

[18] Mazza JRSE, Lambert J, Zunzunegui MV, Tremblay RE, Boivin M, Côté SM. (2017). Early adolescence behavior problems and timing of poverty during childhood: a comparison of lifecourse models. Soc Sci Med.

[19] Russell DW, Clavél FD, Cutrona CE, Abraham WT, Burzette RG. (2018). Neighborhood racial discrimination and the development of major depression. J Abnorm Psychol.

[20] Earnshaw VA, Rosenthal L, Lewis JB (2013). Maternal experiences with everyday discrimination and infant birth weight: a test of mediators and moderators among young, urban women of color. Ann Behav Med.

[21] Perry BL, Harp KL, Oser CB. (2013). Racial and gender discrimination in the stress process: implications for African American women's health and well-being. Sociol Perspect.

[22] Lewis TT, Cogburn CD, Williams DR. (2015). Self-reported experiences of discrimination and health: scientific advances, ongoing controversies, and emerging issues. Annu Rev Clin Psychol.

[23] Fuchs A, Resch F, Kaess M, Moehler E. (2022). Early parenting stress links obstetric complications and child psychopathology in middle childhood in an at-risk sample. J Dev Behav Pediatr.

[24] Krieger N, Williams DR, Moss NE. (1997). Measuring social class in US public health research: concepts, methodologies, and guidelines. Annu Rev Public Health.

[25] Wheeler LA, Updegraff KA, Crouter A (2015). Mexican-origin parents' work conditions and adolescents' adjustment. J Fam Psychol.

[26] Piler P, Kandrnal V, Kukla L (2017). Cohort profile: the European longitudinal study of pregnancy and childhood (ELSPAC) in the Czech Republic. Int J Epidemiol.

[27] Cox JL, Holden JM, Sagovsky R. (1987). Detection of postnatal depression. Development of the 10-item Edinburgh Postnatal Depression Scale. Brit Journal Psychiatry: J Mental Sci.

[28] Liskola K, Raaska H, Lapinleimu H (2021). The effects of maternal depression on their perception of emotional and behavioral problems of their internationally adopted children. Child Adolesc Psychiatry Ment Health.

[29] Lasalvia A, Zoppei S, Van Bortel T (2013). Global pattern of experienced and anticipated discrimination reported by people with major depressive disorder: a cross-sectional survey. Lancet.

[30] Pascoe EA, Richman LS. (2009). Perceived Discrimination and Health: A Meta-Analytic Review. Psychol Bull.

[31] Andreyeva T, Puhl RM, Brownell KD. (2008). Changes in perceived weight discrimination among Americans, 1995-1996 through 2004-2006. Obesity.

[32] Gunzler D, Chen T, Wu P, Zhang H. (2013). Introduction to mediation analysis with structural equation modeling. Shanghai Arch Psychiatry.

[33] Mehmetoglu M. (2018). Medsem: a Stata package for statistical mediation analysis. Intl J Computational Econ Econometrics.

[34] Baron RM, Kenny DA. (1986). The moderator–mediator variable distinction in social psychological research: conceptual, strategic, and statistical considerations. J Pers Soc Psychol.

[35] Iacobucci D, Saldanha N, Deng X. (2007). A meditation on mediation: evidence that structural equations models perform better than regressions. J Consum Psychol.

[36] Zhao X, Lynch JG, Chen Q (2010). Reconsidering Baron and Kenny: myths and truths about mediation analysis. J Consum Res.

[37] Raghunathan TE, Lepkowski JM, Van Hoewyk J, Solenberger P. (2001). A multivariate technique for multiply imputing missing values using a sequence of regression models. Surv Methodol.

[38] Szymanski DM, Stewart DN. (2010). Racism and sexism as correlates of African American women's psychological distress. Sex Roles.

[39] Dias BG, Ressler KJ. (2014). Experimental evidence needed to demonstrate inter- and trans-generational effects of ancestral experiences in mammals. BioEssays.

[40] Brown RH, Eisner M, Walker S (2021). The impact of maternal adverse childhood experiences and prenatal depressive symptoms on foetal attachment: Preliminary evidence from expectant mothers across eight middle-income countries. J Affect Disord.

[41] Lux V, Non AL, Pexman PM, Stadler W, Weber LAE, Krüger M. (2021). A developmental framework for embodiment research: the next step toward integrating concepts and methods. Front Syst Neurosci.

[42] Hou Y, Kim SY, Hazen N, Benner AD. (2017). Parents’ perceived discrimination and adolescent adjustment in Chinese American families: mediating family processes. Child Dev.

[43] Levy JK, Darmstadt GL, Ashby C (2020). The influence of gender-transformative programming on the health and well-being of children and adolescents: a systematic review. Lancet Global Health.

[44] Gupta GR, Oomman N, Grown C (2019). Gender equality and gender norms: framing the opportunities for health. Lancet.

[45] Heymann J, Levy JK, Bose B (2019). Improving health with programmatic, legal, and policy approaches to reduce gender inequality and change restrictive gender norms. Lancet.

[46] Taylor K, Guerin PT. (2019).

[47] Wagman P, Nordin M, Alfredsson L, Westerholm PJM, Fransson EI. (2017). Domestic work division and satisfaction in cohabiting adults: associations with life satisfaction and self-rated health. Scand J Occup Ther.

[48] Křížková A, Vohlídalová M, Pospíšilová K, Maříková H. 2017. Aktuální rozdíly v odměňování žen a mužů v ČR Hloubková analýza statistik a mezinárodní srovnani. [Current differences in the remuneration of men and women in the Czech Republic. In-depth analysis of statistical data and international comparison.] Ministerstvo práce a sociálních věcí, Praha. [Ministry of Labor and Social Matters, Prague]. ISBN 978-80-7421-147-8. https://www.rovnaodmena.cz/www/img/uploads/336b8482.pdf. Accessed 22 May 2022

[49] Schwab K, Eide EB, Zahidi S (2014). http://www3.weforum.org/docs/GGGR14/GGGR_Comp.

[50] Koldinská K. (2015).

[51] Mozny I, Rabusic L. (1992). Unmarried cohabitation in Czechoslovakia. Czechoslovak Sociological Rev.

