# Supplementary material for: Maternal gender discrimination and child emotional and behavioural problems: A population-based, longitudinal cohort study in the Czech Republic
Source: eClinicalMedicine. 2022 Aug 27;53:101627. doi: 10.1016/j.eclinm.2022.101627 (PMC9433606; doi:10.1016/j.eclinm.2022.101627)
Supplement: Supplementary file 1 [file mmc1.pdf]

Supplementary material

Maternal gender discrimination and child emotional and behavioural problems: a population-based, longitudinal cohort study in the Czech Republic

Professor Irena Stepanikova, Sanjeev Acharya, Alejandra Colón-López, Safa Abdalla, Jana Klanova, Professor Gary L. Darmstadt

Table S1: Child strength and difficulties questionnaire (SDQ) scores by gender discrimination at baseline before multiple imputations

	Perceived gender discrimination, baseline				All	
	Yes		No			
Variable (Range)	Mean	SD[1]	Mean	SD	Mean	SD
SDQ score						
7 years (range 0-1.5)	0.51 (n=288)	0.26	0.45 (n=2279)	0.24	0.46 (n=2567)	0.24
11 years (range 0-2.21)	0.75 (n=200)	0.36	0.67 (n=1673)	0.32	0.68 (n=1873)	0.33
15 years (range 0-1.45)	0.39 (n=150)	0.25	0.37 (n=1128)	0.23	0.37 (n=1278)	0.23

[1]SD = standard deviation
